# Techniques for Temperature Monitoring of Myocardial Tissue Undergoing Radiofrequency Ablation Treatments: An Overview

**DOI:** 10.3390/s21041453

**Published:** 2021-02-19

**Authors:** Martina Zaltieri, Carlo Massaroni, Filippo Maria Cauti, Emiliano Schena

**Affiliations:** 1 Department of Engineering, Università Campus Bio-Medico di Roma, Via Alvaro del Portillo, 00128 Rome, Italy; m.zaltieri@unicampus.it (M.Z.); c.massaroni@unicampus.it (C.M.); 2 Arrhythmology Unit, Cardiology Division, S. Giovanni Calibita Hospital, Isola Tiberina, 00186 Rome, Italy; filippocauti@hotmail.it

**Keywords:** temperature measurements, thermocouples, thermistors, fiber bragg grating sensors, fluoroptic sensors, infrared thermometry, magnetic resonance thermometry, ultrasound thermometry, myocardial radiofrequency ablation, cardiac radiofrequency ablation

## Abstract

Cardiac radiofrequency ablation (RFA) has received substantial attention for the treatment of multiple arrhythmias. In this scenario, there is an ever-growing demand for monitoring the temperature trend inside the tissue as it may allow an accurate control of the treatment effects, with a consequent improvement of the clinical outcomes. There are many methods for monitoring temperature in tissues undergoing RFA, which can be divided into invasive and non-invasive. This paper aims to provide an overview of the currently available techniques for temperature detection in this clinical scenario. Firstly, we describe the heat generation during RFA, then we report the principle of work of the most popular thermometric techniques and their features. Finally, we introduce their main applications in the field of cardiac RFA to explore the applicability in clinical settings of each method.

## 1. Introduction

Catheter-mediated radiofrequency ablation (RFA) is the most widely used procedure in the field of cardiac electrophysiology. In fact, since its first application in cardiology in 1987 [1,2,3], RFA has emerged as the key procedure for the treatment of multiple arrhythmias, due to the low mortality and morbidity associated with this practice, together with its high success rate [4].

Myocardial RFA is a minimally invasive technique which exploits high-frequency alternating electrical current to induce irreversible damage in selected myocardial districts through hyperthermia. During RFA, high temperatures of at least 50 °C are reached to cause irreversible damage on the target tissue with consequent cell death. Temperatures equal or above 100 °C should not be attained since are often cause of dangerous complications [5]. In fact, steam popping, tissue perforations and hematic clots upon the catheter tip are the main possible operative drawbacks, since at 100 °C the water contained in the cells undergoes immediate boiling, the blood proteins denature and the tissues surrounding the catheter tip incur drying [6,7,8]. From a macroscopic point of view, RFA produces lesions that are constituted by a central portion of necrotic tissue bordered by a zone of inflamed tissue, in which cellular excitability is zeroed [9].

The shape and dimension of the produced lesions, and consequently the outcome of procedure, are strongly related to the temperature and its history [10]. Therefore, monitoring the temperature increase of the treated tissue during cardiac RFA and, more generally, during all kind of thermal treatments (i.e., microwave ablation (MWA), laser ablation (LA), and high-intensity focused ultrasound (HIFU)), may be of fundamental importance, not only to ensure the success and the safety of the procedures, but also to adjust the progress of the parameters set (e.g., power delivery and treatment time).

Given this need, in the last few decades the effort made by researchers to develop methods for temperature monitoring during ablation procedures with better performance has led to the achievement of several solutions exploiting various technologies.

Fluoroptic sensors [11,12,13,14,15] as well as fiber Bragg grating sensors (FBGs) [16,17,18,19] have been largely employed during LA. Image-based thermometric techniques (i.e., computed tomography (CT) and magnetic resonance imaging (MRI)) have also been investigated and could be promising for use in clinical settings. Thermometry based on CT [20,21,22] and MRI [23,24,25] proved to be the most suitable in this scenario. Thermocouples [26,27,28,29] and thermistors [30,31,32,33] held in probes have also been exploited but can be affected by significant measurement errors [34].

The most commonly used techniques for temperature monitoring during HIFU are non-invasive. Among others, thermometry based on ultrasound [35,36,37,38] and MRI [39,40,41] has been the most exploited. The use of thermal probes embedding fluoroptic fibers [42,43] and thermocouples [44,45,46] was also explored but at the expense of the non-invasiveness, which is one of the main advantage of HIFU.

Temperature monitoring based on MRI [47,48,49,50] and CT [51,52,53] has emerged as valuable technique in the MWA clinical practice. Nevertheless, thermocouples [54,55,56] and thermistors [57,58,59] still represent an alternative solution for MWA temperature detection, although invasive and carrier of possible measurement errors. FBGs’ feasibility have also been investigated in this field [60,61,62].

A similar scenario is encountered for RFA procedures, which are exploited both for the solid tumors’ removal (e.g., liver, lung, pancreatic, and kidney cancers) and for the treatment of cardiac arrhythmias. For the first application, once again MRI thermometry [63,64,65] has played a key role as a non-invasive method, while more rarely infrared (IR) thermometry [66,67] has been used. Moreover, some attempts have been made also through the use of ultrasound-based thermometry [68,69,70] and invasive methods, such as thermocouples [71,72] thermistors [57,73,74,75], and fiber optics [76,77,78,79], but limited to laboratory settings.

Focusing on myocardial RFA, one of the first approaches used, but still widely employed for its ease of use and low costs, is the insertion of thermal probes holding either thermocouples [80] or thermistors [81] directly into the treated tissue. In addition, more than a decade later, fluoroptic probes [82] and FBGs [83] were employed to obtain more reliable and high-resolved temperature measurements, however at increased costs [84]. In the 2000s, the interest in contactless thermometric methodologies led to the exploitation of techniques based on ultrasound tomography [85], IR [86], and MRI [87]. Despite the obvious advantages brought by these approaches (e.g., uncluttered surgical field and lack of additional devices to be managed), to date they are not in widespread usage due to many limitations that technology has not yet managed to overcome [88,89].

In this paper the working principle of RFA is described. Then, an overview of the principal techniques used for measuring the temperature variation which cardiac tissue experiences during RFA procedures is presented. The reported solutions for thermal detection are divided into two main classes: invasive (or contact-based) and non-invasive (or contactless) techniques. For every thermometric solution, a global background of its use in different thermal therapies (i.e., MWA, LA, HIFU and RFA) is shown. The principle of work of each technique is presented, then an accurate focus on the application in myocardial RFA is provided. Moreover, an evaluation on the performances and a comparison between benefits and drawbacks brought by each methodology is reported.

## 2. General Principles of Radiofrequency Ablation 

During RFA procedures, an alternating electrical current at high-frequency is provided to the target tissue by means of a catheter. Specifically, a continuous unmodulated sinusoidal waveform current whose frequency ranges from 350 kHz to 750 kHz is produced by the RF generator. These frequency values are not high enough to induce ventricular fibrillation [5]. The current is delivered between the antenna tip electrode and a ground plate which is located in contact with the patient’s skin with the help of electrical conducting gel. On the tip electrode, called the ablation electrode, the passage of the electrical current is focused, while on the ground plate (also called dispersive or indifferent electrode) minimum current density is ensured thanks to its large area of contact (typically greater than 10 cm^2^) [4].

The formation of the thermal damage (or lesion) is the result of the heating mechanism. Such a process can be considered the outcome of two main contributions: the resistive heating involving the tissue surrounding the tip, and the conductive heat transfer into the underneath layers [90]. Considering the capacitive effects to be negligible, the power delivered into the target tissue during RFA is reported in the equation below:

P = I^2^ R (1)

where I is the current provided, and R the total resistance (i.e., the sum of the resistances relative to the catheter, blood, tissue, and ground plate). Hence, resistive heating process is strictly related to the local power density (p) which, in turn, depends on the current density (j) and R, according to the following equation:

p = j^2^ R (2)

**Figure 1:**
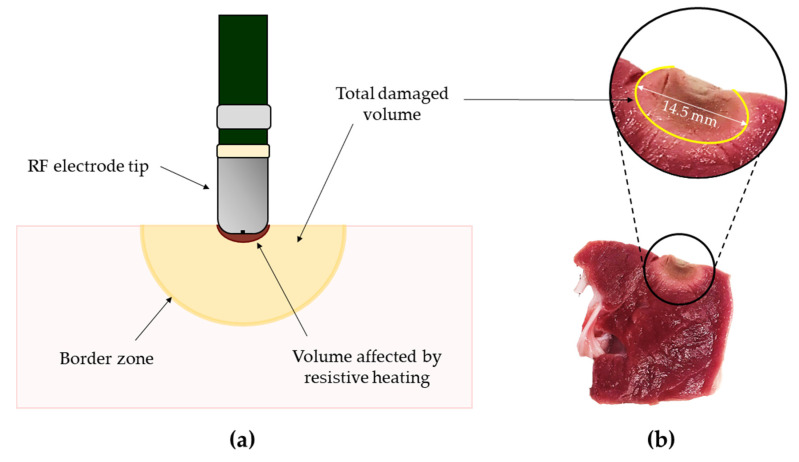
The thermal damage produced in tissues subjected to radiofrequency ablation (RFA) procedures. (**a**) Schematic representation of the total ablated volume of tissue and the resistive heating volume; (**b**) an example of a real thermal damage produced by RFA on myocardial tissue. The total damaged volume (yellow ellipsis) is highlighted. In this specific case, 60 W were delivered for 60 s by means of a 2.5 mm diameter tip exerting 12 gf (i.e., approximately 0.12 N) on the tissue. The produced thermal damage was 14.5 mm in length. The picture is adapted from [83].

Given that j decreases as 1/r^2^, where r is the distance from the catheter application point, p drops within the tissue as 1/r^4^ [91]. Therefore, only a thin layer (that is about 1 mm [4]) of tissue surrounding the tip can be considered to be subjected to the resistive heating action [92]. Instead, the total damaged volume (whose dimensions depend on several factors such as delivered power, treatment time and pressure exerted on the tissue by the tip) is governed by both conductive and convective heating exchanges. In fact, the layers below the antenna exchange conductive heat, while the interaction with flowing blood and tissues at lower temperatures provokes convective cooling. A schematic representation of the thermal damage is shown in Figure 1.

The cells’ destruction and irreversible injury depend on temperature and exposure time [10]. In myocardial tissue, cells’ depolarization with consequent excitability loss starts at about 43 °C and remains reversible until reaching 48 °C for any treatment time. Several in vitro experiments have identified the temperature of about 50 °C as the value at which permanent injury occurs [5]. Moreover, as the temperature increases, the time for reaching cytotoxicity shortens, as described by the Arrhenius’ equation [93,94]:

(3) $$\Omega \;=\;\int_{0}^{t}Ae^{-E/RT\left(t\right)}dt$$

where Ω is the natural log of the ratio of the concentration of the altered tissue state to the original state, *A* the collision frequency, *E* the activate energy, *T*(*t*) the absolute temperature, and *R* is the universal gas constant.

## 3. Invasive Solutions for Myocardial Temperature Evaluation during RFA

In this section, four invasive solutions for temperature evaluation during myocardial RFA are presented. All these methods involve direct contact with the measurement site.

### 3.1. Thermocouples: Working Principle and Application in Myocardial RFA 

Since their first utilization in 1935, thermocouples have been the leading technology for thermal measurements in the field of thermal treatments thank to their small size, robustness, low cost, and reliability [95,96,97]. However, their accuracy is lower than that of some other thermometers (e.g., resistance temperature detector and thermistor).

Although thermocouples are largely used for temperature monitoring during MWA [54,55,56], RFA [71,72], LA [26,27,28,29], and HIFU [44,45,46] for cancer removal, they are also employed in myocardial RFA. Specifically, thermocouples embedded in RF emitting antennas have been widely adopted both in the clinical practice [98,99] and in the experimental field [100,101,102,103]. Nevertheless, such configuration records a single-point measurement which does not provide direct information regarding the temperature of the tissue undergoing RFA. Thus, no work exploiting this configuration will be described in this review which is focused on temperature monitoring of myocardial tissues.

In the following two paragraphs the working principle of the thermocouples, their applications in cardiac RFA as well as their main advantages and drawbacks are described.

#### 3.1.1. Working Principle 

Thermocouples are temperature sensors composed of two junctions of different metal conductors. Their working principle is based on the foundation that two differing conductors forming a circuit and exposed to a thermal gradient produce and electromotive force (emf). This phenomenon is also known as the Seebeck effect [95]. The emf presents a non-linear dependence on the difference between the temperatures experienced by the two junctions. The two metals coupling is chosen in order to guarantee the maximum possible emf, taking into account that the materials must be chemically compatible [104]. Many suitable combinations of conductive materials are available, depending on the need and the field of application. For example, base metals and their alloys are exploited to produce thermocouples for the detection of low and moderate temperatures, so being suitable for cryoablation processes. On the contrary, thermocouples made from platinum, as well as nickel-chromium alloys, are mostly employed in hyperthermal treatments since they can be used in oxidizing environments and are stable at extreme temperatures (up to even 1400 °C and 1100 °C, respectively) [105].

#### 3.1.2. Applications of Thermocouples in Myocardial RFA

Since the use of RFA has prevailed as the leading procedure for the treatment of arrhythmias, many research groups have investigated the relationship between the temperature detected within the tissue and lesion size. Measurement probes holding thermocouples have been widely used for this aim.

In 2000, Cao et al. [80] developed a new system to monitor the temperature trend during myocardial RFA. The custom system was composed of a thermistor fixed at the antenna tip and a temperature probe made by three thermocouples placed inside the tissue. Firstly, the two instruments (one using the thermistor and one using the thermocouples) were statically calibrated. Then their dynamic response was assessed in terms of time constant (it was found to be 0.4 s for the thermistor, and 0.25 s, 0.28 s, and 0.30 s for the three thermocouples) whose values ensured a response fast enough to follow the temperature variation in tissue. It is worth noting that the constant times of the thermocouple-based probe were longer than the stand-alone sensors (i.e., 0.08 s) since the presence of glue and of a low pass filter within the circuit. Explorative trials were performed by means of five RF ablations showing that the proposed system was able to follow the temperature trend inside the organs. One year later, the same group [106] used the aforementioned system to investigate the influence of the cooling effect provoked by the blood flowing in the heart chambers on the lesion formation. Specimens of bovine heart were inserted into a water bath at 37 °C in presence of a pump (Model 180, Precision Scientific, Winchester, VA, USA) to simulate the flow inside the heart chamber (typically ranging between 0 L·min^−1^ and 6 L·min^−1^). The custom three-thermocouples system (T-type thermocouples, Physitemp Instrument Inc., Clifton, NJ, USA) was inserted directly below the ablation site to evaluate the temperature within the inner myocardium in three measurement sites. The three thermocouples were fixed at different depths (i.e., 0.9 mm, 2 mm, and 3 mm). Several ablations were performed by means of a Blazer II emitting catheter (EP Technologies, San Jose, CA, USA) holding a thermistor at the tip at the target temperatures of 60 °C and 80 °C for three different values of flow rate (i.e., 0 L·min^−1^, 1 L·min^−1^ and 3 L·min^−1^). Larger lesions were observed for higher flowrate. Moreover, temperature trends within the tissue reached the plateau faster under higher flow rate. Both these effects could be explained by the temperature compensation mechanism played by the RF generator. In fact, the greater the cooling caused by the saline flow, the greater the increase in the delivered power to ensure the reaching of the target temperatures. Also, the maximal temperatures detected by the thermocouples decreased consistently with the distance from the emitting source. For example, fixed 60 °C of target temperature, the thermistor placed into the antenna detected 60 °C while temperatures of approximately 53 °C, 50 °C, and 46 °C were recorded by the three thermocouples (placed at a depth of 0.9 mm, 2 mm, and 3 mm, respectively).

Starting from the work of Petersen and co-authors [101] exploring the relationship between the lesion size and the heat diffusion inside the tissue, in 2003 Eick et al. [107] investigated the differences resulting from the use of traditional catheters vs. irrigated ones. Temperature controlled RFA were performed with both an irrigated (RF Sprinklr, Medtronic EPSystems) and a non-irrigated (RF Marinr, Medtronic EPSystems, Minneapolis, MN, USA) electrodes on in vitro swine ventricular samples in presence of 20 mL·min^−1^ saline flow. The target temperatures (measured in the electrode) were set at 50 °C, 60 °C and 70 °C, and the tissue contact forces ranged between 0.04 N and 0.67 N. Also, a thermocouple was placed 2 mm under the ablation site. The differences between the temperatures measured by the thermocouple integrated into the antenna and that placed in the tissue were evaluated: tissue temperature after 30 s of irradiation was 42 ± 6 °C higher than that measured into the electrode for irrigated procedures. On the contrary, for non-irrigated RFA the tip temperature exceeded the tissue temperature of 33 ± 2 °C. These findings reinforce our choice to omit the studies focused only on temperature monitoring in the electrode since it is not representative of the actual tissue temperature.

In 2010, Halm et al. [108] evaluated the occurrence of esophageal injuries during myocardial RFA exploiting an esophageal probe equipped with five thermocouples. In this study, 185 patients were subjected to left atrial RFA by means of an irrigated catheter (Agilis, St Jude Medical, St Paul, MN, USA) with 30 s of treatment time and temperature control protocol (i.e., 48 °C of target temperature at the electrode tip and 50 W of maximal power). The temperature probe was located into the esophagus, orthogonally to the RF radiation. Esophageal ulcers, whose dimensions were 7 ± 3.2 mm, have been observed in 27 patients whose intraluminal temperatures reached values above 41 °C. Statistical results showed that for each 1 °C of temperature variation, the esophageal injury dimension increased by a factor of 1.36. This interesting study highlights the importance of temperature monitoring in the tissue surrounding the probe (in this case in the esophagus) as a tool to prevent injuries.

In 2017 Halbfass et al. [109] explored the incidence of the esophageal injuries occurrence after myocardial RFA performed with and without esophageal thermal probes devoted to local temperature control. 80 patients were subjected to left atrial RFA performed with the following setting: 35 W of maximum power delivery, target temperature at the electrode tip of 43 °C, at least 20 s of treatment time and contact force ranging from 10 gf (corresponding to approximately 0.098 N) to 35 gf (corresponding to approximately 0.343 N). The esophageal temperature was monitored in 40 patients (Group 1) by means of a temperature probe (S-Cath™, CIRCA Scientific, LLC, Englewood, CO, USA) embedding 12 thermocouples, while the remaining part (Group 2) was subjected to a traditional procedure. In Group 1, when temperature higher than 39 °C were detected, the power delivery was stopped. No esophageal perforation occurred in the study, but post-treatment esophageal asymptomatic lesions were detected in both the groups (i.e., 7.5% and 10% in Group 1 and 2, respectively). However, the maximum lesion size found in Group 2 (i.e., 35 mm) was greater than that found in Group 1 (i.e., 10 mm). The study demonstrated a low occurrence of severe and extended esophageal damages (ulcers) in accordance with the use of the thermocouple-based temperature probe located in the esophagus lumen.

Summing up, thermocouples are one of the most commonly used technologies for temperature investigation in the field of cardiac RFA. Their deployment typically implies their encapsulation in needles or shields to promote chemical isolation from the surrounding biological environment. These sensors are well known, and have a small size, low price and adequate performance relative to the application context.

However, the conductive metallic components which constitute thermocouples may interact with the incident RF field, thereby inducing measurement artifacts [110,111]. These effects can be partially overcome by preferring small diameters probes inserted into the tissues with the long axis orthogonal to the incident electromagnetic field [97]. In addition, the presence of one or more thermocouples probes within the operating field might obstruct the already crowded area, so limiting their usage in the clinical scenario.

### 3.2. Thermistors: Working Principle and Application in Myocardial RFA

Thermistors are widely used for temperature monitoring during all kind of hyperthermic treatments. Such sensors are a viable alternative to thermocouples due to their good metrological characteristics. Compared to thermocouples, they can yield better performance as present slightly higher accuracy (i.e., better than 0.3 °C). Low cost, small size and fast time response are additional advantages.

Thermistors have found ample space for use in MWA [57,58,59], RFA [57,73,74,75], and LA [30,31,32,33].

Concerning cardiac RFA, thermistors have been widely used, both held into the antenna tip [112,113,114,115,116,117] and inserted in thermal probes. Nevertheless, since the temperature information provided by thermistors embedded in the RF emitting electrodes is solely related to the tissue’s surface, only studies employing thermal probes placed in the inner layers of the treated tissues will be described in this work, as previously done for the thermocouples.

#### 3.2.1. Working Principle

Thermistors are resistance temperature sensors as they are composed of a semiconductor material (generally oxides of nickel, manganese, iron, copper or doped ceramics) whose resistance changes according to the temperature variation. The relationship between the thermistor’s resistance and the detected temperature is non-linear, as shown in the following equation [118]:

(4) $$R_{T}=R_{0}\;\exp\;\left[1-B\;\left(\frac{1}{T}-\frac{1}{T_{0}}\right)\right]$$

where R_T_ and R_0_ are the resistances at the measured temperature *T* and at the reference temperature *T*_0_, respectively. *B* is the thermal constant related to the specific material the thermistor is made of.

#### 3.2.2. Applications of Thermistors in Myocardial RFA

As for thermocouples, thermistors have played a crucial role in temperature monitoring during cardiac RFA, both in clinical and research fields.

In 2006, Kovoor et al. [81] investigated the in vivo effects of RFA on healthy tissues versus scarred tissues. Previously, acute myocardial infarction was induced on 5 mongrel dogs to produce tissue scarification. After tissue healing, a bipolar catheter (ZENCOR MF1, Zencor, Sidney, Australia) delivered RF energy for 60 s and at target temperature of 90 °C. A thermistor was embedded in the antenna tip, while another five needles holding thermistors were inserted to monitor temperature in the tissue surrounding the electrode; 50 ablations were made, 25 on the normal tissue and 25 on the scarred one. Data showed no differences in impedance values between the two tissues as well as the power required for the ablations (i.e., average of 1.8 W). Also, the temperature profiles obtained by the six thermistors did not differ from normal to scarred myocardium and, as a consequence, the produced lesions presented similar sizes. This study dispelled the common belief that RFA would be less effective on injured tissues than on healthy ones. 

Redfearm et al. [119] in 2005, explored the feasibility of using esophageal temperature as a truthful predictor of the success of myocardial RFA. In [119] a thermistor-based esophageal temperature probe (Mallinckrodt Mon-a-therm embedding Thermistor 400 Series) was placed in the esophagus of 15 patients undergoing RFA at 50 W or 60 W, for 20 s or 30 s of treatment time. The probe was placed at three different levels of the esophagus: at the midline (3 patients), on the right (2 patients) and on the left (10 patients) wall of the lumen. Treatments were performed in the posterior wall of the left atrium including the pulmonary veins. The maximal temperature increment was 3.7 °C (starting from a baseline temperature of 37 °C) detected by the probe that was closer to the pulmonary veins. Nevertheless, only probes that were less than 1 cm from the ablation site could appreciate a significant rise in temperature, suggesting that such an approach was still too immature to be safely exploited in clinical practice.

In 2007 Rodrìguez et al. [120] presented a whole novel approach. An agar phantom was designed to simulate the biological features of the human heart and esophagus. Static and dynamic calibrations of an esophageal temperature probe based on a thermistor (ER400-9, Respiratory Support Products Inc., Tijuana, Mexico) were performed. The time constant found for the probe (τ_P_), the exposed thermistor (τ_M_), and the thermocouple (τ_T_) were approximately 8.0 s, 1.5 s, and 40 ms, respectively. The probe was placed inside an esophageal tube 6.5 mm deep into the phantom, together with an exposed thermistor and a thermocouple fixed on its surface, in correspondence with the RF antenna. The probe was placed at different distances from the ablation site (at a constant depth of 6.5 mm and different longitudinal distances ranging from 0 mm to 20 mm), while the thermocouple was placed just beneath the tip at 6.5 mm in depth. For each position five ablations were executed for 60 s at the target temperature at the electrode tip of 55 °C. The esophageal probe measured lower temperature peaks than thermocouple (39.6 ± 1.1 °C vs. 48.3 ± 1.9 °C), with minimum values for the greater distances from the RF antenna (i.e., 20 mm). Such temperature underestimation was probably the result of both the high value of τ_P_ compared to τ_T_ and the bigger distance of the thermistor from the emitting source compared to that of the thermocouple. This solution can be considered useful to increase the safety of myocardial RFA procedures if used in conjunction with other methodologies.

To reassume, the application of thermistors in the field of cardiac RFA presents an evolution comparable to that of thermocouples for similar characteristics and performances [95]. Low cost, small size and fast time response, as well as better accuracy are the main advantages related to this sensor. On the contrary, thermistors’ measurements can be affected by noise as a result of the coupling between the connecting wires holding metallic components and the RF radiation [97].

### 3.3. Fluoroptic Sensors: Working Principle and Application in Myocardial RFA

Fluoroptic sensors are part of the fiber-optic sensors (FOSs) macrofamily. Their notable metrological characteristics, such as wide temperature measurement (i.e., from −25 °C to 300 °C), high accuracy (i.e., 0.2 °C), rapid response to thermal variation (τ in the order of µs), small size (i.e., up to 0.1 mm in diameter) and inertness to biological environments [121,122], has made them suitable for clinical research exploring the heating diffusion within tissues. In addition, the immunity to electromagnetic fields ensures no interaction with external fields.

This last feature promoted attempts to use fluoroptic sensors in MW [123,124] and RF [125,126,127,128] treatments. Moreover, these sensors have been largely exploited also during HIFU [42,43] and LA procedures [11,12,13,14,15].

Because of the invasiveness of the approach, this technology has been used in cardiac RFA thermography more for ex vivo and in vivo experiments on animals than for clinical application on patients.

#### 3.3.1. Working Principle

The operating principle of fluoroptic sensors exploits the natural sensitivity of some fluorescent species to temperature variations. These sensors are generally composed by thermosensitive fluorescent particles such as magnesium fluorogermanate activated with tetravalent manganese (Luxtron Technologies, Santa Clara, CA, USA), thulium, alexandrite or other rare-earth elements. Such species are inserted into a conventional fiber optic and placed at its edge. Once excited by a pulsed light generated from a source, the phosphor layer produces a fluorescent signal that propagates back in the fiber to a detector. Fluorescence is the result of an emission of photons in response to the excitation of the fluorescent layer. The emitted signal intensity (I_P_) decays following an exponential trend, as reported in the following equation:

(5) $$I_{P}\;=\;I_{0}\;e^{\frac{-t}{\tau }}$$

where I_0_ is the intensity at the initial instant, *t* is the time, and *τ* is the decay time which strictly depends on the temperature to which the fluoroptic particles are exposed [129]. As consequence, by evaluating *τ* it is possible to go back to the temperature information [121,130].

#### 3.3.2. Applications of Fluoroptic Sensors in Myocardial RFA

Fluoroptic fibers have been mainly devoted to temperature measurement in RF treatments performed on animal myocardium, both ex vivo and in vivo.

In 2005, Wood et al. [82] exploited fluoroptic fibers to investigate the relationship between microbubble formation and temperature increase during cardiac RFA. Four fluorometric temperature probes (STB, Luxtron, Inc., Santa Clara, CA, USA) were inserted into isolate portions of porcine myocardium at different depth (i.e., from 8 mm to 10 mm). The tissues were placed in saline, together with an ultrasound probe devoted to the detection of microbubbles formation. The RFA was performed with different power and treatment time settings to yield the formation of different types of microbubbles (i.e., type 1—scattered microbubbles and type 2—continuous microbubble formation). Results showed that, regardless of the type of microbubbles, their presence always indicated vapor formation and attainment of temperatures far above those typically used in myocardial RFA. In fact, type 1 and type 2 occurred at temperatures of 81.0 °C ± 5.0 °C and 91.4 ± 8.2 °C, respectively. Steam pops onset at 105.9 ± 7.5 °C. This suggested that the temperature monitoring in the inner layer may help in predicting the formation of unwanted injuries much more than power values, tissue impedance, and superficial tip temperature.

A year later, Thyer et al. [131] investigated the feasibility to apply cooling saline irrigation to hearts undergoing RFA in order to prevent coronary artery damage; 17 entire ovine hearts were placed in saline bath and subjected to 60 s RF treatment (15 W of maximum power delivery and 50 °C of maximal temperature). Two fluoroptic probes (700, Luxtron, Inc., Santa Clara, CA, USA) were placed within the coronary artery directly under the ablation site and 15 mm below. The samples were divided in two groups: the first was exposed to the cooling effect of a saline solution delivery into the coronary artery, while the second was not. Data proved the validity of the method as the temperature measured by the fluoroptic sensors, were higher in the absence of the cooling system (i.e., 54.6 °C maximum peak without cooling vs. 23.6 °C maximum peak with cooling).

Watanabe et al. [132] in 2010 explored the cooling effect induced on the myocardium by RF catheters incorporating saline irrigation systems. Intramyocardial temperature was detected in 10 hearts of anesthetized mongrel dogs by means of four fluoroptic probes (3100, Luxtron, Inc., Santa Clara, CA, USA) inserted at several distances from the emitting RF antenna. The treatment lasted 90 s at the target temperature of 40 °C in temperature-controlled modality. Compared with previous studies, the use of saline-cooled antennas resulted in a lower incidence of steam pops and slower power rise.

Certainly, among all the FOSs, fluoroptic sensors are the prevailing technology in the literature for temperature monitoring during myocardial RFA. These sensors present two possible configurations: embedded into probes or, more rarely, without any shield. The presence of the probe causes a significant increment of the response time. The quality of the measurements, the immunity to electromagnetic fields, small size, flexibility, and the multiplexing capability have led over the years to an ever-greater use of this type of sensor. However, compared to thermocouples and thermistors, fluoroptic fibers present significantly higher costs. Once again, the invasiveness of the method limited its application to the experimental field. 

### 3.4. Fiber Bragg Gratings: Working Principle and Application in Myocardial RFA

FBGs are to date one of the most widely used technology in the area of the modern thermal sensing exploiting optical fibers. FBGs allow providing high accurate temperature measurements (e.g., 0.1 °C) and short response time (in the order of µs) [133]. It is worth noting that these sensors have multiplexing capabilities that allows the measurement of temperature in multiple sites using a single optical fiber. Also, the most recent manufacturing methodologies (i.e., drawing tower fabrication [134] and point-by-point laser inscribing [135]) allow the production of sensors whose length varies from centimeters up to few millimeters for high-resolved measurements for temperature maps reconstruction.

Such technology has been largely employed in RFA [76,77,78,79], LA [16,17,18,19] and MWA [60,61,62] treatments. Recently, FBGs have been tested also in HIFU procedures [136].

Nevertheless, the fragility of the fibers and the motion artifact caused by the organs’ movements, to date have limited the use of FBGs in the clinical practice.

#### 3.4.1. Working Principle

A FBG is a periodic refractive index modulation in the core of a small section of fiber optic. The FBG works as a notch filter; in fact, once a full spectrum light produced by an optical interrogator propagates along the fiber, a small portion of spectrum whose peak is centered around a specific wavelength (hereafter the Bragg wavelength, λ_B_) is reflected back to the source. λ_B_ depends on the effective refractive index (η_eff_) and the grating period (Λ), as indicated in the equation below [137]:

(6) $$I_{P}\;=\;I_{0}\;e^{\frac{-t}{\tau }}$$

External perturbations occurring in temperature variations as well as strain effects cause a shift (Δλ_B_) in the λ_B_ described as follows:

(7) $$\frac{{\Delta \lambda }_{B}}{\lambda_{B}}\;=\;(1\;-\;\rho_{\varepsilon })\Delta \varepsilon \;+\;(\alpha_{f}+\;\xi_{f})\Delta T$$

where ΔT and Δε are variations in temperature and strain, respectively. $\rho_{\varepsilon }$, $\alpha_{f},\;\xi_{f}$ are in the respective cases the photo-elastic coefficient, the thermal expansion coefficient and the thermo optic coefficient related to the fiber core. When Δε is negligible, Δλ_B_ can be considered result of ΔT only and the FBG can be exploited as a thermometer.

#### 3.4.2. Applications of Fiber Bragg Gratings in Myocardial RFA

To date, the use of FBG sensors for thermal trends reconstruction in the context of cardiac RFA is limited to a very recent study.

A first attempt was made by Zaltieri et al. [83] in 2020. The authors moved a step into this field by using FBGs for multipoint temperature measurements. Two specimens of freshly excised swine myocardium were placed in saline bath at controlled temperature. Two optical fibers (FiSens GmbH, Braunschweig, Germany) embedding seven FBGs each (1 mm long with 2 mm edge-to-edge distance, thermal sensitivity of 0.01 nm∙°C^−1^) for a total of 14 measurement sites, were inserted orthogonally to the tissue’s surface and at different distances from the emitting irrigated electrode (FlexAbilityTM Ablation Catheter Sensor EnabledTM, Abbott Medical, MN, USA). RFA was performed at 50 W and 60 W of power delivery for 60 s of treatment time, exerting 12 gf (corresponding to approximately 0.118 N) on the tissue by the catheter. The temperature profiles showed a delay in temperature rising for the deeper sensors, in accordance with the heating mechanism of biological tissues. Higher values of temperature increment (24 °C and 33.5 °C for 50 W and 60 W trials, respectively) were measured by the sensors closest to the surface and belonging to the array placed at the shorter distance from the antenna tip. Moreover, larger lesions were obtained for the higher power value. However, the thermal damage expanded more in width than in length, suggesting that myocardial tissue has a preferential direction of heat diffusion. This study laid new basis for the development of multipoint thermal maps based on FBG sensing which can allow a three-dimensional reconstruction of tissue temperature.

In the last few decades, although there are high costs (mainly related to the interrogation system) compared to thermocouples and thermistors, FBGs have found a large range of use for temperature monitoring in the field of ablation treatments [84]. Good thermal sensitivity and accuracy, short response time, multiplexing capability, immunity to electromagnetic fields are the main advantages of this technology. On the contrary, the fragility and the cross-sensitivity to strain (caused for example by cardiac and respiratory activity) may limit the use of this methodology in in vivo trials on the myocardium.

### 3.5. Short Summary on Invasive Solutions for Myocardial Temperature Monitoring during RFA

Invasive techniques provide real-time, single-point or multi-point temperature detection within the inner layers of myocardial tissues undergoing RFA.

Thermocouples and thermistors have often been applied indistinctly as they present similar metrological features for the range of temperatures required in cardiac RFA. Both the technologies are well known in clinical and research applications, present adequate accuracy, fast response, small size, and robustness. However, these systems are not free of limitations. In fact, thermocouples and thermistors offer single-point measurements, unless more sensors are inserted into the treated tissue, but making the treatment area extremely cluttered. Moreover, these sensors are prone to measurement errors caused by the interaction of metal components with the RF electromagnetic field. 

Fluoroptic sensors present good characteristics such as high accuracy, rapid thermal response, reduced dimensions (unless when inserted within a probe) and immunity to electromagnetic radiations. However, increased costs compared to the previous technologies and fragility are the main weakness.

The main advantage of FBGs over all the other mentioned techniques is the multiplexing capability. Thus, their use allows for providing multipoint measurement by embedding several sensors within a single optical fiber. In addition, also this technology resents no disturbance from electromagnetic fields. On the contrary, FBGs present high costs (mainly related to the interrogation dispositive) and cross-correlation with strain. The fibers’ fragility is an ulterior drawback.

A summary of the applications and the features related to the invasive solutions presented is reported in Table 1.

## 4. Non-Invasive Solutions for Myocardial Temperature Evaluation during RFA

In this section the most popular solutions for non-invasive temperature monitoring in cardiac RFA are reported: MRI, ultrasound imaging and IR imaging are presented, together with their main applications. Although thermometry based on CT imaging has been used in several thermal treatments [20,21,138], in this review we do not describe this technique because it has not already been applied to myocardial RFA. The numerous studies on non-invasive techniques are fostered by two features that can overcome the main hurdles in the use of invasive techniques in clinical practice: their non-invasiveness and the possibility to reconstruct a temperature map of the entire treated volume.

### 4.1. Magnetic Resonance Imaging: Working Principle and Application in Myocardial RFA

Starting from the early 1990s, the adoption of MRI to detect in real-time tissue temperature during hyperthermia treatments has come to fruition for in vivo applications [139,140,141,142,143,144,145].

Good temporal resolution (i.e., less than 2 s) and high accuracy are just some of the benefits brought by this technology [146].

MRI-based thermometry exploits the sensitivity of numerous MR-related parameters (i.e., water proton density, T_1_ and T_2_ relaxation times of water protons, water diffusion, magnetization transfer and water proton resonance frequency, PRF) to temperature variation [147]. Several studies have proven the feasibility of such methods for temperature detection and compared their performance [148,149], but it has not been possible to draw global conclusions, since the good outcome of each technique depends on the type of application and the features of the tissue to be monitored.

To date, it is reasonable to state that the PRF-based technique exhibits high precision with respect to methodologies exploiting T_1_ and water diffusion, as PRF does not show tissue-type dependency. Furthermore, it presents high sensitivity (about −0.01 ppm·°C^−1^) and linear dependence for temperatures ranging from −15 °C to 100 °C [88].

MRI thermometry has found discrete application mainly in LA [23,24,25], and HIFU [39,40,41] ablation treatments, as well as MWA [47,48,49,50] and RFA [63,64,65] for oncologic treatments.

Currently, the most commonly used technique in myocardial RFA procedures is the PRF-based, even if such an approach is still considered a niche activity [88]. The main applications in cardiac RFA are shown below.

#### 4.1.1. Working Principle

As specified in the previous paragraph, in the field of cardiac RF procedures, the most commonly used approach for MR-thermometry is the one based on PRF (hereafter PRF shift thermometry, PRFST).

In 1966 Hindman et al. [150] first observed the shift in PRF due to temperature variations. Such shift in PRF is defined as the change that a nucleus experiences with respect to a reference nucleus. The PRF of a specific nucleus depends on the magnetic fields it is subjected to (*B_loc_*). As reported in Equation (10) [151], *B_loc_* is function of the external magnetic flux density (*B*_0_) and the shielding constant (*s*) which depends on the chemical environment:

(8) $$B_{loc}=\;\left(1-s\right)B_{0}$$

Considering that the electrons held into the hydrogen nuclei shield the water dipole from *B*_0_, the resonance frequency of the nucleus results as follows [151]:

(9) $$\omega \;=\;\gamma B_{loc}=\;\gamma \left(1-s\right)B_{0}$$

where γ is the hydrogen gyromagnetic ratio. 

In a biological tissue, the electronic shielding process to which the hydrogen nucleus is subjected is more effective the more the water molecule is free from hydrogen bonds with the neighboring molecules. In fact, the hydrogen bridges cause a decrease in the shielding as they provoke a distortion in the electronic configuration. Moreover, the temperature increase also influences hydrogen bonds as makes the inter-molecular linkages more labile so improving the shielding effect. Since PRF is temperature-dependent, it is possible to trace the thermal information through two approaches: the spectroscopic imaging [139] and the phase shift mapping [152]. For the first method, the temperature variation is detectable as the shift of peaks of the water spectrum. However, the most exploited is the second one, thanks to which the ΔT occurring within the tissue subjected to heating can be determined as described in the following equation [151]:

(10) $$\Delta T\;=\;\frac{\varphi \left(T\right)-\varphi \left(T_{0}\right)}{\gamma \;\alpha_{1}\;B_{0}\;TE}$$

where φ(*T*) and φ(*T*_0_) are the phases of the image in the current and initial instants at the corresponding temperatures *T* and *T*_0_, respectively. α_1_ represents the PRF change coefficient and *TE* is the echo time.

#### 4.1.2. Applications of Magnetic Resonance Imaging in Myocardial RFA

Starting from the first decade of the 2000 s, the use of PRFST has aroused increasing interest in cardiac RFA.

In 2010, Kolandaivelu et al. in [87] have verified the possibility of using PRFST on myocardium subjected to RFA by minimizing movement artifacts through post-processing reduction techniques. Six mongrel dogs underwent temperature-controlled treatments (maximum temperatures at 60 °C and 80 °C at the electrode tip) and were thermally inspected by a 1.5 T-MRI scanner (Espree, Siemens Medical Systems, Erlangen, Germany). To operate a correction of the displacements related to the heart beating, pre-and post-heating images were registered, then the post-heating image was shifted, the two images were subtracted and converted in a ΔT map. Results showed that myocardial areas in which temperatures of 50 °C were detected well correlated with the size of the produced lesion.

A further step forward was made a year later by Hey et al. [153] who assessed the performance of three blood suppression methods combined with two MR sequences in the optimization of cardiac thermometry. PRFST by means of the 3 T-MR system Achieva (Philips Healthcare, Best, The Netherlands) during in vivo experiments in 8 volunteers with no presence of heating mechanism were performed, while a parallel imaging gradient echo with and without echo-planar imaging (EPI) readout acceleration was applied. Also, three blood suppression techniques were tested. The best results were achieved by using the EPI readout together with the inflow saturation blood suppression (temperature stability of 2 °C, resolution of 3.5 × 3.5 × 8 mm^3^, and a temporal resolution of one heartbeat).

Starting from this, in 2012 de Senneville et al. [154] deepened the topic by improving temporal resolution and volume coverage of the acquired images. At first, the feasibility of the PRFST was assessed on 10 volunteers in normal breathing conditions using 1.5 T-MRI Achieva (Philips Healthcare, Best, The Netherlands). The images were then subjected to post-processing to minimize motion artifacts. Two sheep were subjected to myocardial RFA and the tissue’s temperature was monitored by using PRFST with update rate of 1 Hz. The mean standard deviation of the temperature trend was 3.6 ± 0.9 °C, 2.8 ± 0.9 °C and 4.1 ± 0.9 °C for the left ventricle, the septum and the heart-lung interface, respectively. This suggests that PRFST has adequate performance for application in monitoring temperature during myocardial RFA.

In 2017, several works have been published by Toupin and coworkers [155,156,157]. In both [155,156], the authors tried to overcome the impossibility of evaluating the dimension of the lesion resulting from cardiac RFA by considering the thermal dose absorbed by the tissue as a predictor. PRFST was performed by using a 1.5 T-MR system Avanto (Siemens Healthcare, Erlangen, Germany) on 5 volunteers in normal conditions. Results showed an uncertainty of 1.5 °C. The same protocol was exploited on 3 sheep subjected to cardiac RFA. In [155] a high correlation factor (R = 0.87) was obtained between the real lesion sizes and the dimension estimated through PRFST data. In [156] an improved MRI pipeline with on-line reconstruction combined with rapid imaging was employed. Such an approach improved thermal imaging output without significant loss of temporal resolution, making it suitable for real-time measurements. Starting from these two works, in [157] a novel optical-flow motion algorithm was incorporated to the already presented MR pipeline to minimize the motion artifact contributions. The assessment was performed on 9 healthy volunteers, on a healthy sheep, and on a phantom. Compared to the previous H&S algorithm, results showed faster responses and enhanced themperature accuracy (<1.5 °C of temperature error, algorithm computational time of 25 ms per image).

Thanks to the high sensitivity, no outcome dependency from the specific tissue, and linear dependency with the temperature in the range explored in cardiac RFA, PRFST is increasingly being used in clinical research. Nevertheless, the diffusion of such an approach in real clinical scenarios is slowed by the high costs and the need to position the patient into the MRI machine together with the necessity of using MRI-compatible surgical tools. Moreover, the wide occurrence of motion artifacts related to cardiac and respiratory movements afflict the measurements. However, some methods to minimize this effect have been proposed [88,89,158].

### 4.2. Ultrasound Imaging: Working Principle and Application in Myocardial RFA

Ultrasound imaging is an attractive method based on mechanical waves which does not require time-consuming post-processing procedures also allowing real-time mapping.

Starting from 1979, early investigations assessing the feasibility of applying ultrasound imaging for temperature detection in biological tissues were carried out by several research groups [159,160,161,162,163,164]. Ultrasound-based thermometry has found certain applications in the field of HIFU [35,36,37,38], and RFA [69,70] therapies, mainly on hepatic tissues, and to a lesser extent during LA [165,166] and MWA [167]. Despite the large use of ultrasound imaging in clinical practice, only a few studies have been conducted in the field of myocardial RFA.

#### 4.2.1. Working Principle

Ultrasounds are mechanical waves whose frequencies exceed the upper limit of the human auditory range which is set at 20 kHz [168]. The ultrasound imaging thermometry technique consists in delivering high-frequency waves into the target body district by means of a delivery probe. The interaction between waves and tissues produces a backscattered signal which is transmitted to the probe and read by it, while the remaining energy continues its transmission along the material. By examining the characteristics of the backscattered/transmitted waves, it is possible to reconstruct information regarding the target tissue. In particular, such a methodology is based on the examination of three different effects occurring in biological materials experiencing heating: (I) attenuation of the transmitted signal, (II) power variation, and (III) echo-shift in the backscattered signal [161]. To date, the most commonly exploited approach in the medical field is the third.

The time delay, *t*, of a reflected wave at temperature *T*_0_ depends on the position (z) and the speed of sound in a given material at *T*_0_ temperature (*c*_0_ (*T*_0_)), as shown in the following equation:

(11) $$t\left(T_{0}\right)\;=\;\frac{2z}{c_{0}\left(T_{0}\right)}$$

The ΔT experienced by material causes two main phenomena: thermal expansion and change in the speed of sound. These effects provoke a shift in the travel-time of the reflected signal. Considering that the thermal expansion contribution can be treated as negligible compared to the one brought by the change in speed of sound, the relation between ΔT at z position and the time delay can be indicated as follows [169]:

(12) $$\Delta T(z)\;=\;\frac{c_{0}}{2\left(\alpha -\beta \right)}\cdot \frac{\delta t\left(z\right)}{\delta z}$$

where *t*(z) is the time shift at z position along the propagation axis, *c*_0_ is considered the speed of sound at the tissue temperature before the heating process, α is the thermal expansion coefficient and *β* is a coefficient which represents the speed of sound variation during heating. *β* varies linearly as the temperature increases, up to about 50 °C [170,171].

By means of a motion algorithm which evaluates the Δt in each consecutive backscattered wave, it is possible to evaluate the temperature evolution in time in different points along the propagation axis and in contiguous beams. This process permits a 2D temperature map to be obtained.

#### 4.2.2. Applications of Ultrasound Imaging in Myocardial RFA

At present, only one study evaluating thermal distribution into myocardial tissues undergoing RFA are present in the literature.

In 2001 Seo et al. [85] presented a new method to detect cell necrosis temperature. Experiments were performed both ex vivo on porcine specimens and in vivo on Yorkshire pigs’ hearts. A custom RFA antenna embedding an ultrasound delivery probe (GE Vivid 7, GE Healthcare, Horten, Norway) was used to simultaneously deliver RF and transmit and read the ultrasound waves for real-time temperature measurements. By calculating the thermal strain evolution in time and considering the slope of this curve, it was possible to assess that 50 °C temperature occurred when the slope curved reached the plateau value, both for ex vivo and in vivo trials. This threshold may be useful to estimate the damaged volume, since cell injury occurs when tissues reach about 50 °C [172]. The main difference between in vivo and ex vivo experiments was that the thermal strain plot showed a more uncertain and less defined pattern, due to motion artifacts.

The use of such technology for cardiac RFA thermal investigations purposes has been encouraged by the easy supply of ultrasound probes in hospitals and research centers [173]. Unfortunately, this approach is affected by motion artifacts caused by the natural physiological activity of the organ undergoing RFA. These effects can be minimized by post-processing, again raising the computational costs [174]. In addition, at temperatures above 50 °C the change in the speed of sound is less sensitive, thereby providing unrealistic measurements [170,171]. However, the information on a specific temperature threshold (i.e., approximately 50 °C) retrieved by this decrement of sensitivity may be beneficial to estimate the amount of damaged volume.

Summing up, ultrasound imaging for cardiac RFA temperature monitoring has not yet achieved wide use in clinical practice.

### 4.3. Infrared Imaging: Working Principle and Application in Myocardial RFA

IR thermography is a contactless technique that permits temperature information to be obtained from the measured wavelength spectrum emitted by all the objects. The use of this methodology permits to get high-resolved bidimensional images of the whole radiating surface in real-time.

As thermal imaging technology, it has found application in the medical field primarily in clinical diagnosis of tumors in different body districts [175,176,177,178]. More rarely, IR imaging has been used to monitor minimally invasive hyperthermic therapies, principally due to the lack of inner thermal information. Few studies are found in the literature, and those are mainly for ablating therapies involving HIFU [179,180] and RF [67].

Starting from the first decade of the 2000 s, this technique has been investigated to assess its goodness in evaluating temperature trends of myocardial tissues subject to RFA.

#### 4.3.1. Working Principle

IR thermometry is based on the principle that all bodies having a higher temperature than absolute zero emit electromagnetic radiation (called infrared or thermal radiation) whose wavelength ranges from 0.75 µm to 1000 µm [181]. Depending on whether a range of values the emitted wavelength belongs to, the radiation will then be called near infrared (i.e., from 0.76 µm to 1.5 µm), medium infrared (i.e., from 1.5 µm to 5.6 µm) or far infrared (i.e., from 5.6 µm to 1000 µm), respectively [182].

The thermal emission of the human tissues can be explained by introducing the concept of the blackbody, which is an abstract body capable of absorbing and emitting the full spectrum of radiation. According to Planck’s law, the radiation emitted by the blackbody can be described as follows [183]:

(13) $$\frac{dE\left(\lambda ,T\right)}{d\lambda }\;=\;\frac{2\pi hc^{2}\lambda^{-5}}{\exp\left(\frac{hc}{\lambda kT}\right)-1}$$

where $\frac{dE\left(\lambda ,T\right)}{d\lambda }$ is called spectral exitance, *h* and *k* are the Plank’s and Boltzmann’s constants, respectively, *c* is the speed of light and *T* represents the absolute temperature of the blackbody.

By integrating the Planck’s law between *λ* = 0 and *λ* = ∞, the Stefan–Boltzmann law is obtained [183]:

(14) $$E\;=\;\sigma T^{4}$$

where E represents the total emissive power, σ is the Stefan–Boltzmann constant (which is equal to 5.67 × 10^−8^ W·m^−2^·K^−4^) and *T* is the body’s absolute temperature. For real materials, the ε constant representing the emissivity of the surface at defined *λ* and *T* is introduced. Thus, Equation (14) becomes:

(15) $$E\;=\;\varepsilon \sigma T^{4}$$

where ε is always <1. ε is experimentally determined and depends on the emitting angle and the temperature of the radiating body, as well as the physical (i.e., geometry and roughness) and chemical (i.e., contamination) features of the emitting surface [184]. Fixed ε and σ, and knowing the emissivity spectrum of a specific tissue of the human body at a given temperature, it is possible to trace the thermal information (i.e., *T*) by comparing the measured *λ* with the reference one. For instance, a freshly excised human epicardium at 40 °C emits in the wavelength from 3 µm to 5 µm with an ε of about 0.86 [185].

#### 4.3.2. Applications of Infrared Imaging in Myocardial RFA

Since the early 2000 s, IR technology has been used in cardiac RFA to delimit the contours of the induced lesions and characterize the proprieties of myocardial tissue [186,187], but it was only in the second decade that this technology took hold in this field to provide thermal feedback.

In 2011 Wood et al. [86] proposed a novel approach based on IR imaging to detect the tissue temperature at the border of the lesion. Fifteen RF deliveries (power values from 20 W to 25 W and treatment time from to 6 s to 240 s) in ex vivo myocardial porcine specimens submerged in saline bath at 38 °C were performed in the presence of an IR thermal camera (T400, Flir, Inc., Danderyd, Sweden) with accuracy of ±2%. The temperature map was overlapped with a picture of the specimen collected by an optical camera. The lethal isotherm was considered to be the isotherm which corresponded to the contours of the lesion shown by the picture. The mean value of the lethal isotherm was calculated as 60.6 °C (ranging from 58.1 °C and 64.2 °C), suggesting that the temperature reported in literature at which there is irreversible cell destruction (i.e., 50 °C) overestimates the lesion dimension.

A totally different approach was presented in three different studies published in 2018 in which luminal esophageal temperature detection via IR imaging was used for thermal monitoring during cardiac RFA [188,189,190]. In [188] an IR thermography catheter, IRTC (produced by Securus Medical Group, Inc, Cleveland, OH), was inserted into the esophagus of 16 volunteers who received RF deliveries at 35 W. Daly and coworkers analyzed the thermal trends for each patient and obtained a spatial temperature gradient of 2.3 ± 1.4 °C·mm^−1^ and a temperature change rate of 1.5 ± 1.3°C·s^−1^. Visible esophageal lesions happened only in patients who experienced luminal temperatures greater than 50 °C. In [189] Hummel et al. aimed at define a limit esophageal temperature that might be used as a cutoff value to interrupt RF delivery. Real-time IR thermometry was performed (at a sampling frequency of 1 Hz through the use of IR probes (Securus Medical Group, Inc, Cleveland, OH, USA) placed into the esophagus in intimate connection with the left atrium of the volunteers; 46 °C and 50 °C were fixed as maximum values the esophagus lumen could experience; once those temperatures were reached, the RFA delivery was stopped. The study revealed that no esophageal injuries occurred for temperatures below 50 °C, thus suggesting that an esophageal temperature cutoff of 50 °C could help improve cardiac RFA efficiency by preventing undesired damages. In [190] Borne et al. presented a pilot study in which 16 volunteers were enrolled to be subjected to low power (i.e., 20 W) RFA while being monitored via an IRTC. In 10 volunteers temperatures above 40 °C were detected and, once again, esophageal injuries were observed only for luminal temperatures above 50 °C.

All these studies paved the way for a potential use of luminal esophageal temperature via IR imaging to improve the safety in cardiac RFA procedures.

High-resolution bidimensional images of the whole radiating myocardial surface can be obtained in real-time through the usage of IR thermometry. Nevertheless, this technique is not devoid of disadvantages. The main drawbacks are: lack of information regarding the inner body temperature (as the wavelength emission only belongs to the superficial layers of the body), high costs, large influence of movement artefacts (which have made it difficult to use for thermal monitoring of moving bodies), and background reflectance (which may affect the emission) [191].

### 4.4. Short Summary on Non-Invasive Solutions for Myocardial Temperature Monitoring during RFA

The presented non-invasive techniques allow the thermal pattern reconstruction of the myocardial tissue subjected to RFA avoiding direct contact with the organ. MRI, ultrasound imaging and IR imaging ensure no insertion of additional tools inside the treated area, thus reducing overcrowding in already crowded operating fields with experimental or surgical equipment. Also, all the presented imaging methodologies did not use ionizing radiation. Moreover, the lack of contrast fluids intake makes them suitable for repeated applications.

Among other approaches, MRI is the most exploited. The use of PRFST offers high precision, good temporal resolution, linear relationship with temperature variation from −15 °C to 100 °C and no outcome dependency from the specific tissue. On the contrary, such a technique is deeply affected by the motion artifacts caused by the cardiac and respiratory activities. Algorithms devoted to artifact elimination could be exploited, but at the expense of computational costs. Moreover, the need to operate within the MRI room, thus to use MRI-compatible surgical tools, limits its use in clinical practice. Also, the costs (of both MR scanners and specific sequences for thermometry) are higher compared to IR- and ultrasound-based imaging.

Regarding ultrasound thermometry, ultrasound probes are significantly less expensive than MRI scanners and also readily available in hospital environments. Good accuracy and spatial resolution can be achieved by carefully choosing both a performing motion algorithm and a proper ultrasound pulse. However, once again, this entails an incrementation of computational costs. The main drawbacks related to this technology are: the large occurrence of motion artifacts caused by the organs’ physiological activity (i.e., cardiac and breathing activities) and the measurement errors due to the change in the speed of sound in tissues exposed to temperatures greater than 50 °C.

Finally, IR imaging provides a real-time bidimensional color-coded map for easy interpretation. To date, this technique has been mostly exploited for preventing unwanted injuries of anatomical structure surrounding the myocardium (e.g., esophageal lumen), instead of reconstructing the cardiac temperature. This is because the IR system (which is a catheter) needs to be placed in correspondence of the measurement site. Nevertheless, this method is highly affected by the surrounding environment as instruments and operators could invade the scanning field thus distorting the measurements. Moreover, no information regarding the inner layers of the treated tissues is provided by the use of this technique.

A summary of the features related to the non-invasive solutions presented is reported in Table 2.

## 5. Discussion and Conclusions

From the first cardiac RFA application in 1987, it became clear that temperature control was a requirement of primary importance.

In this work an overview of the most popular and promising systems devoted to temperature monitoring in cardiac tissues undergoing RFA was presented. The methodologies have been divided into two main categories: invasive and non-invasive solutions. For each methodology, the working principle, the performance, the field of application with a focus on cardiac research and clinical implementation, and the main pros and cons were presented.

To date, thermocouples and thermistors embedded into RF antenna tips are the technologies most used in clinical practice. However, in this configuration, the sensors do not return information on the internal temperature of the treated tissue, but rather a punctual measurement at the point of contact between the myocardial surface and the tip. This system is useful for monitoring the parameters set on the RF generator (such as delivered power and treatment time) to avoid the formation of ulcers, steam pops and blood clots, but it is not suitable to determine what are the effects of RFA within the tissue. To accomplish this task, temperature probes holding thermocouples and thermistors have been commonly used both in the clinical (i.e., placed into the esophageal lumen) and in the research (i.e., directly inserted into the myocardium) fields. The main drawbacks are represented by: single point measurements (unless additional probes are inserted, however, making the working area very crowded); and measurement errors due to the interaction between the RF electromagnetic field and the metallic components from which thermocouples and thermistors’ connecting wires are made.

On the contrary, fiber-optic sensors (i.e., FBGs and fluoroptic sensors) offer a viable alternative. Specifically, FBGs seem to be the most promising solution since this approach allows multipoint temperature detections to be performed with high accuracy, adequate spatial resolution, and short time-response, as well as not being affected by the presence of electromagnetic radiation. On the contrary, FBGs present higher costs compared to thermocouples and thermistors.

The main issue led by the usage of all the presented contact-based technologies during in vivo trials is their invasiveness, as direct insertion into the tissue is required.

The need to find technologies for non-invasive temperature monitoring in myocardial tissue subjected to RFA has led scientists to focus on the improvement of imaging techniques such as MRI, ultrasound tomography, and infrared imaging. With respect to the invasive methods, these approaches aim at presenting temperature maps of the entire target tissue.

Despite its high costs, MRI is the most commonly used among the imaging methodologies, as provides high-accurate maps with good acquisition speed. Moreover, the PRFST allows temperature detection overcoming tissue-type dependency.

Ultrasound-based and infrared-based approaches are less exploited and appear mainly in pilot studies. High spatial resolution can be achieved for ultrasound imaging, albeit worsening the signal transmission in the tissue. Good resolution is also obtainable with infrared imaging, but the temperature information is retrieved only on the superficial layers of the tissues.

The main advantages offered by the presented non-invasive technologies are: contactless measurements (with no cluttering of the operating table by means of additional surgical instruments), no exploitation of ionizing radiation and no requirement of contrast liquids intakes. On the contrary, image-based thermometry is often affected by motion artifacts caused by natural cardiac activity. Many research groups have focused their attention on the search for methods aiming to solve this limitation. However, in most cases the minimization of motion artifacts is at the expense of the computational cost. A summary of the main features that characterize each invasive and non-invasive technique is reported in Table 3.

In conclusion, several methods for temperature monitoring during cardiac RFA have been presented and their main benefits and burdens have been discussed. Currently, no approach can be considered free from drawbacks, so the choice of a particular solution depends on the needs and the circumstances related to the individual case.

In clinical practice, the currently used techniques do not allow real temperature estimation to be performed inside the myocardial tissue. In fact, thermocouples and thermistors held into the emitting antennas provide temperature at the antenna tip which only gives indirect information for the adjustment of the treatment parameters. Also, infrared probes placed in the esophagus lumen are used solely to avoid unwanted injuries.

In the clinical scenario, despite its limits, MRI-based thermometry might be the most promising technique for myocardial thermal monitoring, given the already positive results present in the literature.

In the research context, invasive systems are exploited for in vivo and ex vivo experiments to characterize the effects of cardiac RFA by means of internal temperature analysis. Moreover, such competences may help in optimizing myocardial RFA procedure on patients, also making the process safer. Among other techniques, FBGs present enormous potential and could have a major impact in analyzing the influence of different parameters (i.e., temperature, power delivered, treatment time and force exerted by the catheter) on the mechanism of lesion formation. By means of this new awareness, a relevant contribution would be given on the performance evaluation of new RF delivery devices, as well as on the design guidance of these tools.

Finally, it is possible to conclude that at present finding the optimal measurement system for myocardial temperature monitoring during RFA is still an open challenge.

## Figures and Tables

**Table 1:** Application and Features of the Presented Invasive Solutions Devoted to Temperature Monitoring of Myocardial Tissue Undergoing RFA.

First Author, Year, Ref.	Measurement System	Type of Experiment	Features
Cao et al., 2000−2001 [80,106]	Probe embedding 3 thermocouples	RFA on ex vivo bovine myocardium in saline bath	Time constant = 0.08 s (but approximately 0.2 s when inserted in the proposed probe)
Eick et al., 2003 [107]	Probe embedding a single thermocouple	RFA on ex vivo swine myocardium in saline bath	
Halm et al., 2010 [108]	Esophageal probe embedding 5 thermocouples	185 patients undergoing myocardial RFA	
Halbfass et al., 2017 [109]	Esophageal probe embedding 12 thermocouples	80 patients undergoing myocardial RFA	
Redfearm et al., 2005 [119]	Esophageal probe embedding a single thermistor	15 patients undergoing myocardial RFA	
Kovoor et al., 2006 [81]	5 probes embedding a single thermistor	5 mongrel dogs undergoing myocardial RFA	Time constant = 0.2 s
Rodrìguez et al., 2007 [120]	Esophageal probe embedding a single thermistor + single exposed thermistor	Agar phantom	Time constants = 8.0 s for the esophageal probe and 1.5 s for the exposed thermistor
Wood et al., 2005 [82]	4 fluoroptic probes	RFA on ex vivo swine myocardium in saline bath	
Thyer et al., 2006 [131]	2 fluoroptic probes	RFA on ex vivo ovine myocardium in saline bath	
Watanabe et al., 2010 [132]	4 fluoroptic probes	10 mongrel dogs undergoing myocardial RFA	
Zaltieri et al., 2020 [83]	2 fiber optics embedding 7 FBGs ^1^ each	RFA on ex vivo swine myocardium in saline bath	Sensing length = 1 mm Thermal sensitivity = 0.01 nm∙°C^−1^

^1^ FBGs: fiber Bragg grating sensors.

**Table 2:** Application and Features of the Presented Non-Invasive Solutions Devoted to Temperature Monitoring of Myocardial Tissue Undergoing RFA.

First Author, Year, Ref.	Type of Sensor	Model (In Vivo, Ex Vivo, In Vitro)	Features
Kolandaivelu et al., 2010 [87]	MRI ^1^	6 mongrel dogs undergoing myocardial RFA	FOV ^3^ = 220 × 165 mm Resolution = 256 × 192 Slice thickness = 4 mm
Hey et al., 2011 [153]	MRI ^1^	8 patients undergoing myocardial RFA	FOV ^3^ =350 × 350 × 8 mm^3^ Resolution = 3.5 mm^2^ Slice thickness = 8 mm
De Senneville et al., 2012 [154]	MRI ^1^	2 sheep undergoing myocardial RFA	FOV ^3^ = 250 × 166 mm Resolution = 2.6 mm^2^ Slice thickness = 7 mm
Toupin et al., 2017 [155]	MRI ^1^	5 patients + 3 sheep undergoing myocardial RFA	FOV ^3^ = 225 × 225 mm^2^ Resolution = 1.8 × 1.8 × 4 mm^3^ Slice thickness = 3 mm
Ozenne et al., 2017 [156]	MRI ^1^	10 patients undergoing myocardial RFA	FOV ^3^ = 180 × 180 mm^2^ Resolution =1.6 × 1.6 × 4 mm^2^ Slice thickness = 3 mm
Toupin et al., 2017 [157]	MRI ^1^	9 patients + 1 sheep undergoing myocardial RFA + 1 agar phantom	FOV ^3^ = 180 × 180 mm^2^ Resolution = 1.6 × 1.6 × 3 mm^3^
Seo et al., 2001 [85]	Ultrasound Imaging	RFA on ex vivo swine myocardium in saline bath + in vivo on swine myocardium	Imaging depth = 5 mm/10 mm Imaging width = 45° Frame rate = 32 Hz/1 Hz
Wood et al., 2011 [86]	IR ^2^ Imaging	RFA on ex vivo swine myocardium in saline bath	Sensitivity = 0.005 °C Accuracy = ±2%
Daly et al., 2018 [188]	IR ^2^ Imaging	16 patients undergoing myocardial RFA	Dimension = 3 mm of diameter Resolution = 0.1 °C
Hummel et al., 2018 [189]	IR ^2^ Imaging	34 patients undergoing myocardial RFA	Dimension = 3.5 mm of diameter
Borne et al., 2018 [190]	IR ^2^ Imaging	16 patients undergoing myocardial RFA	Dimension = 3 mm/6 mm of diameter

^1^ MRI: magnetic resonance imaging. ^2^ IR: infrared. ^3^ FOV: field of view.

**Table 3:** Summary of the Features of the Presented Invasive and Non-Invasive Solutions Devoted to Temperature Monitoring of Myocardial Tissue Undergoing RFA.

Technology	Features
Thermocouples	Invasive; single point measurement; accuracy of approximately 1 °C; robust; well-known technology; almost constant sensitivity in a wide range of T; adequate dynamic response considering the application of interest; wide measuring interval; potential presence of measurement artifacts.
Thermistors	Invasive; single point measurement; accuracy better than 0.3 °C; robust; well-known technology; high sensitivity but can significantly decrease for high T; adequate dynamic response considering the application of interest; wide measuring interval; potential presence of measurement artifacts.
Fluoroptic Sensors	Invasive; single point measurement; accuracy up to 0.2 °C; fragile; adequate dynamic response considering the application of interest; wide measuring interval; immunity to electromagnetic interference.
FBGs	Invasive; multi-point measurement with resolution even better than 1 mm; accuracy even higher than 0.1 °C; fragile; adequate dynamic response considering the application of interest; wide measuring interval; constant sensitivity in a wide range of T; immunity to electromagnetic interferences; potential presence of motion artifacts.
MRI	Non-invasive; 3D distribution of T; sensitivity up to −0.01 ppm·°C^−1^; constant sensitivity for T from −15 °C to 100 °C; no tissue dependency in case of PRFST ^1^; temporal resolution better than 2 s; motion artifacts.
Ultrasound Imaging	Non-invasive; 3D distribution of T; easy supply in clinical settings; high T resolution at high computational costs; motion artifacts; decrease in sensitivity for T up to 50 °C.
IR Imaging	Non-invasive; 2D color-coded T map; no T information regarding the inner layers; measurement affected by the surrounding environment.

^1^ PRFST: proton resonance frequency shift thermometry.
